# Injury Patterns in Resuscitated Non-Traumatic Cardiac Arrest Patients—A Comparative CT Analysis Between Automated Chest Compression Devices

**DOI:** 10.3390/diagnostics16081179

**Published:** 2026-04-16

**Authors:** Simon Viniol, Lennart Scholand, Alexander König, Susanne Betz, Michael Scheschenja

**Affiliations:** 1 Clinic of Diagnostic and Interventional Radiology, University Hospital Marburg, Philipps-University Marburg, Baldingerstrasse, 35043 Marburg, Germany; 2 Department of Emergency Medicine, University Hospital Marburg, Philipps-University Marburg, Baldingerstrasse, 35043 Marburg, Germany

**Keywords:** cardiac arrest, chest compression devices, injury patterns, CT imaging, patient safety

## Abstract

**Objectives**: The aim of this study was to determine differences in injury types and frequencies between piston-based and band-based automated chest compression devices in patients with non-traumatic out-of-hospital cardiac arrest (OHCA) at a German cardiac arrest center. **Methods**: This retrospective single-center study assessed resuscitation-related injuries in OHCA patients using protocol-based early whole-body CT scans at hospital admission. CT scans were reviewed independently by two reviewers blinded to the compression device used. Between May 2015 and September 2021, all patients resuscitated from non-traumatic OHCA, treated with a mechanical chest compression device, and showing stable return of spontaneous circulation (ROSC) until CT examination according to the institutional standard operating procedure for all OHCA patients were included. Patients were categorized by compression device type, and group differences were analyzed using the Chi-square test and Mann–Whitney U test. In addition, patient-level incidences of rib fracture types were calculated, and risk ratios with corresponding 95% confidence intervals were used to compare rib fracture patterns between groups. A *p*-value of <0.05 was considered statistically significant. **Results**: Among 71 patients, 32 received band-based and 39 piston-based treatment. Both groups were comparable in resuscitation duration, body constitution, and gender ratio, although the band-based group was older. Thoracic injuries predominated, with rib fractures representing the most frequent injury pattern (64/71, 90.1%). The median number of rib fractures per patient was 10 (IQR 8–12) in the band-based group and 9 (IQR 7–12) in the piston-based group. The band-based group had significantly more liver lacerations (5/32, 15.6% vs. 0/39, 0%; *p* = 0.01) and displaced rib fractures (117 vs. 87; *p* = 0.046; patient-level RR = 1.43, 95% CI 1.06–1.93). **Conclusions**: In this observational study of a CT-based cohort of OHCA patients with stable ROSC, the band-based device was associated with significantly higher frequencies of liver lacerations and displaced rib fractures than the piston-based device. These findings should be interpreted as hypothesis-generating and may support further evaluation of device-specific injury profiles in future studies.

## 1. Introduction

Cardiac arrest (CA) continues to challenge healthcare systems around the world because of its still low survival rate of 8.8% at hospital discharge in patients with out-of-hospital cardiac arrest (OHCA) [1]. In Europe the incidence of CA ranges from 67 to 170 individuals per 100,000 inhabitants annually. Cardiopulmonary resuscitation (CPR) can be attempted or continued by professional medical personnel in 50–60% of these cases, translating to 19 to 97 interventions per 100,000 inhabitants [2]. The wide range of these numbers likely reflects differences in registry methodology, case definitions, and emergency medical service systems across countries.

The outcome of these patients depends largely on rapid emergency care and on the delivery of high-quality CPR during the period without spontaneous circulation. High-quality chest compressions therefore are essential for maintaining coronary and cerebral perfusion during resuscitation. Manual compressions remain the clinical standard. However, maintaining guideline-consistent compression depth, rate, and minimal interruptions can be difficult in real-world conditions, particularly during prolonged resuscitation, transport, or invasive procedures. Consequently, mechanical chest compression devices have become increasingly prevalent in many emergency medicine service systems.

These automated chest compression devices generate compressions either by applying a vertical piston-based force to the sternum or by a load-distributing band (LDB) mechanism that constricts the thorax in a semi-circumferential manner [3]. Current international resuscitation guidelines, such as the European Resuscitation Council Guidelines 2025, do not recommend mechanical CPR devices for routine use in all CA patients, primarily because randomized trials have not demonstrated consistent survival benefit compared with high-quality manual CPR and because device deployment may introduce interruptions. Nonetheless, these guidelines acknowledge that mechanical CPR can be reasonable in specific clinical scenarios in which high-quality manual compressions are difficult to provide reliably or pose safety risks, such as during transport, in the catheterization laboratory, or during prolonged resuscitation including bridging strategies (e.g., extracorporeal CPR pathways), provided interruptions related to device placement are minimized [4].

While mechanical chest compression devices may help maintain CPR quality under challenging conditions, chest compressions themselves can also cause injuries. Common resuscitation-related injuries include rib fractures, sternal fractures, thoracic complications, and visceral injuries, some of which may affect the prognosis of these critically ill patients [5,6,7,8]. Because different compression systems apply force in different ways, they may also differ in the type and frequency of associated injuries. Investigating these differences is crucial for advancing the efficacy and safety of CA treatment, providing a basis for evidence-based recommendation when using automated chest compression devices and ultimately reducing the occurrence of resuscitation-related injuries. Despite the widespread use of mechanical CPR devices, comparative data on device-specific injury patterns remain limited, particularly in survivors assessed by early contrast-enhanced whole-body CT (WBCT). Most published studies compare mechanical with manual CPR, whereas direct comparisons between different mechanical compression systems are less common. In addition, many studies on resuscitation-related injuries are based on autopsy findings in non-survivors, which represent a different clinical spectrum and may overrepresent severe injury phenotypes. Consequently, early CT-based injury assessment in survivors remains underrepresented in the current literature [9].

Therefore, the aim of this study was to comparatively evaluate device-specific injury patterns and frequencies between piston-based and band-based mechanical chest compression devices in resuscitated patients using early WBCT at a German Cardiac Arrest Center (CAC).

## 2. Materials and Methods

This retrospective single-center study was conducted in a German CAC with urban and rural catchment areas. The study has received approval from the local ethics committee. For applicable items, it was reported in accordance with the STROBE recommendations for observational studies [10]. Data were collected of patients admitted between May 2015 and September 2021. WBCT was routinely performed immediately after hospital admission in all OHCA patients with stable ROSC as part of a standardized post-resuscitation diagnostic pathway, aiming to detect both potential causes of arrest and resuscitation-associated complications within a clinically actionable timeframe. This approach aligns with contemporary post-resuscitation care strategies [11,12,13].

**Figure 1:**
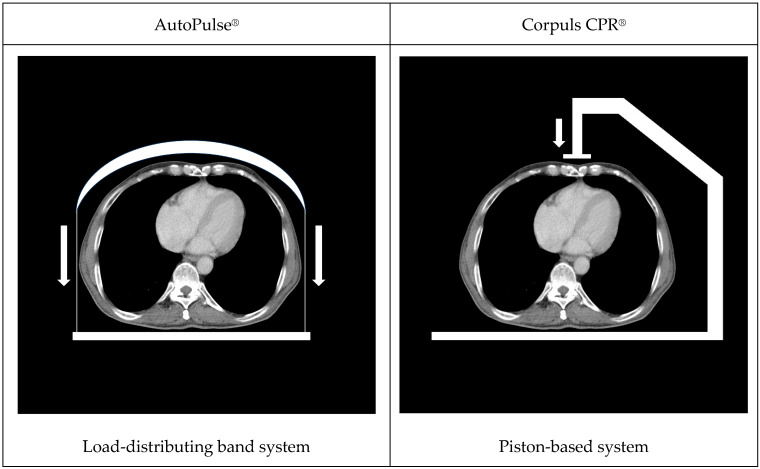
Schematic illustration of thoracic compression device function.

Consecutive patients of all age groups with non-traumatic OHCA who were treated with a mechanical chest compression device during resuscitation, achieved stable return of spontaneous circulation (ROSC) until CT examination, and underwent WBCT immediately after hospital admission were included in this study. Devices were operated by emergency service personnel who had been trained specifically in the use of these devices. Chest compressions were initiated manually and subsequently continued using a mechanical compression device in all cases. During the study period, Corpuls CPR^®^ (GS Elektromedizinische Geräte G. Stemple GmbH, Kaufering, Germany), a piston-based system, and AutoPulse^®^ (Zoll Circulation, Chelmsford, MA, USA), an LDB-based system, were used as chest compression devices (Figure 1).

All included patients were examined within 6 h after admission using a 64-slice detector row CT scanner (Definition AS^®^, Siemens Healthineers, Forchheim, Germany) using a standardized CT protocol. Detailed CT acquisition and reconstruction parameters are provided in the Supplementary Material.

Patients were divided in groups according to the device used. WBCT of the patients were examined for fractures of the ribs, sternum, and spine, as well as for bleeding, pneumothorax, lung contusions, haemorrhagic pericardial and pleural effusion, hematomas in the mediastinum and chest wall, injuries to the liver and spleen. Lacerations were graded according to American Association for the Surgery of Trauma (AAST) [14]. Rib fractures were further categorized into incomplete fractures, complete but non-dislocated fractures, dislocated fractures and multifragmentary fractures. An incomplete fracture was characterized by an abnormal angle in the bone cortex without clear fracture lines on either side. The assessed findings were considered resuscitation-associated injuries in the clinical context of non-traumatic cardiac arrest, while differentiation from clearly pre-existing or unrelated findings was attempted where possible. To compare body habitus between the study groups, we measured chest circumference and presternal subcutaneous fat thickness at the lower third of the sternum, corresponding to the area of maximal force application. These parameters were chosen because thoracic dimensions and soft tissue coverage may influence force transmission and potentially affect resuscitation-related injury patterns as confounding factors. The CT scans were evaluated by two independent reviewers blinded to the compression device used. One reviewer had 8 years of experience in radiology, whereas the second reviewer was a junior reader who had been specifically trained on separate cases before study assessment.

Clinical data was obtained from the hospital’s emergency protocols and the local Radiological Information System (Orbis, Dedalus Healthcare Systems Group, Bonn, Germany). The collected data included utilization of a chest compression device and sex, age, duration of CPR as possible confounding factors. CPR duration was categorized as <1 h and ≥1 h for descriptive purposes.

Continuous variables were tested for normal distribution using the Kolmogorov–Smirnov Test. Comparative statistical analysis of the study groups was conducted using the Chi-square (χ^2^) test for categorical variables and the Mann–Whitney U (MWU) test for continuous variables. Interrater reliability across overall injury assessment between the two reviewers was assessed using Cohen’s kappa. In cases of discrepant findings between the reviewers, a consensus diagnosis was reached after joint discussion, and this consensus assessment was used for all subsequent analyses. Comparisons of rib fracture subtypes between groups were performed at the fracture level. Additionally, incidences of specific rib fracture types per patient were calculated and risk ratios (RRs) with corresponding 95% confidence intervals (CIs) were computed to quantify the strength and direction of associations between device-groups on a patient level. For CPR-duration, analyses were performed on available data. No imputation was undertaken. Statistical significance was assumed at a *p*-value of <0.05. A formal adjustment for confounders and correction for multiple testing was not applied, due to the small sample size and exploratory hypothesis-generating nature of the study. All statistical computations were executed using IBM SPSS Statistics (IBM SPSS Statistics, version 29, Armonk, NY, USA).

## 3. Results

During the study period, 457 patients with non-traumatic OHCA, stable ROSC, and subsequent WBCT were identified. Of these, 71 patients had received mechanical chest compression and were included in the analysis, including 32 patients (45.07%) in the LDB group and 39 patients (54.93%) in the piston-based group. Among these, 52 were male (73.24%) and 19 were female (26.76%), with an average age of the study population of 58.75 ± 14.49 years (youngest 15 years, oldest 85 years) (Table 1). There were no significant differences between the groups regarding gender, duration of chest compression and patient constitution, as assessed by chest circumference and presternal adipose tissue. In both groups, resuscitation durations most commonly exceeded 60 min. Missing CPR duration data were observed in 13 patients in the LDB group (40.61%) and 9 patients in the piston-based group (23.08%). However, age differed significantly between the groups and therefore represented a relevant baseline imbalance. Patients in the piston-based group were younger, with a mean age of 55.41 years, compared with 62.81 years in the LDB group (*p* = 0.03).

Both groups exhibited injuries following chest compression, predominantly in the thoracic region. Overall, rib fractures represented the most frequent injury pattern and were identified in 64/71 patients (90.1%), with a median of 10 (IQR 8–12) rib fractures per patient in the LDB group and 9 (IQR 7–12) rib fractures per patient in the piston-based group. Across the total study population, 27 cases of pneumothorax (38.0%) were identified, including two cases of tension pneumothorax in each group. In total, 35 mediastinal hematomas (49.3%) and 7 haemorrhagic pericardial effusions (9.9%) were detected. None of the mediastinal hematomas were classified as space-occupying, and none of the haemorrhagic pericardial effusions were categorized as tamponade. There was a high level of agreement between both reviewers, with a Cohen’s kappa coefficient of 0.73 (*p* < 0.01), indicating substantial interobserver agreement [15].

With regard to non-rib-fracture injuries, most findings were comparable between the two groups. However, liver lacerations occurred significantly more often and exclusively in the LDB group (5/32; 15.6%) than in the piston-based group (0/39; 0%) (*p* = 0.01) with a moderate effect size. Of these liver lacerations, four were classified as grade I and one as grade II according to the American Association for the Surgery of Trauma (AAST). No statistically significant differences were observed between the two groups in the frequency of active bleeding, haemorrhagic pericardial effusion, sternal fracture, pneumothorax, lung contusion, mediastinal hematoma, thoracic wall hematoma, or spine fracture (Table 2).

**Figure 2:**
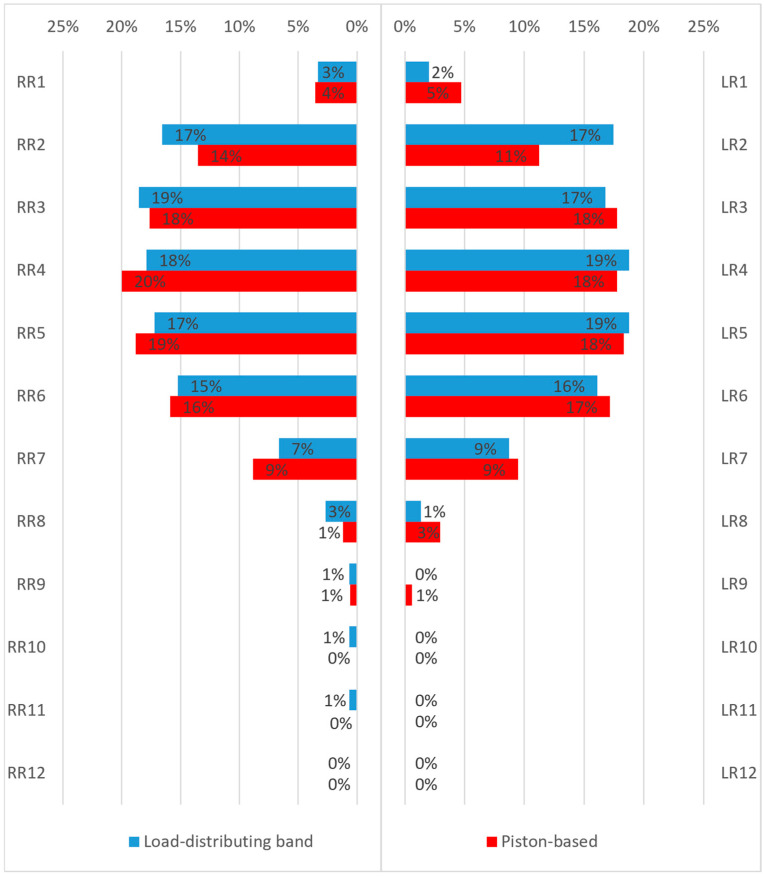
Relative distribution of rib fractures by rib level and side in both device groups. RR indicates right rib and LL indicates left rib. Overall, the distribution pattern was broadly similar between groups, with a tendency toward relatively more frequent involvement of cranial rib levels in the load-distributing band group.

Rib fractures were categorized as displaced, non-displaced, and incomplete fractures. Displaced rib fractures were significantly more frequent within the LDB group than the piston-based group (*p* = 0.046) with a patient level RR of 1.43 (95% CI 1.06–1.93), whereas non-displaced and incomplete rib fractures did not differ significantly between the groups. Table 3 presents fracture-level counts, whereas Table 4 summarizes patient-level incidences and corresponding risk ratios. The higher count of displaced rib fractures in the LDB group did not translate into statistically significant differences in the frequency of pneumothorax, bleeding, hemorrhagic pleural and pericardial effusions, lung contusions, or thoracic hematomas. Figure 2 illustrates the incidence and distribution of rib fractures across both groups.

## 4. Discussion

Independently from device manufacturers, this study examines the differences in injury patterns and severity following chest compression between two automated chest compression devices, an LDB-based and a piston-based system, in a real-world setting of a CAC in Germany. This comparison is of particular interest because the two devices differ in their mechanism, and both are commonly used.

Our findings indicate that both types of devices were associated with injuries, and that most injury categories were broadly comparable between the two devices. Only two endpoints, namely liver lacerations and displaced rib fractures, differed significantly between groups, both occurring more frequently in the LDB group. Importantly and contrary to current suggestions [16], the higher rate of displaced rib fractures in the LDB group did not translate into statistically significant differences in radiographically evident downstream thoracic complications such as pneumothorax, hemorrhagic pleural effusions, hemorrhagic pericardial effusions, mediastinal hematoma, or lung contusions in this cohort.

The design differences between the two systems may plausibly account for the observed outcomes. The larger force-distributing area of the LDB device means that more ribs, and depending on body habitus and band positioning, potentially upper abdominal structures such as the liver, may be within the effective area of compression (Figure 1). This could explain the higher incidence of displaced rib fractures and liver lacerations observed in the LDB group. In addition to the device’s general mechanism, deployment conditions and positioning accuracy are likely relevant mediators of injury risk, particularly for LDB systems, where more caudal positioning could theoretically increase transmission of force to the upper abdomen. However, these considerations remain speculative. Several factors may explain why a higher proportion of displaced rib fractures did not translate into higher rates of pneumothorax or hemorrhagic pleural/pericardial effusions on early CT. Fracture displacement may be an imperfect surrogate for pleural or pulmonary injury or the limited sample size may have reduced statistical power to detect moderate between-group differences in these complications.

Recent evidence confirms that CPR-associated injuries are common overall, with rib fractures being the most frequently reported finding. A systematic review and meta-analysis reported high rates of rib fractures and substantial overall injury burden across non-traumatic cardiac arrest cohorts, while emphasizing wide heterogeneity between studies and detection methods [7,9,17]. This heterogeneity is directly relevant to interpreting device-specific findings across literature: studies differ in inclusion criteria (survivors vs. non-survivors), timing and modality of diagnosis (CT vs. autopsy), and definitions of injury severity (e.g., any rib fracture vs. displaced fractures). Many studies on chest compression devices have been funded by manufacturers and focused primarily on clinical out-comes or usability, rather than systematically analyzing device-associated injuries [18,19,20,21]. Additionally, they often compare mechanical with manual compressions rather than comparatively between devices [22]. Direct evidence on device-specific injury differences remains limited. However, previous studies have suggested that injury profiles may differ between mechanical compression systems. In a randomized safety trial, excess serious or life-threatening visceral damage could not be excluded for an LDB-system compared with manual chest compressions, whereas this signal was not observed for a piston-based system [23]. The signal of visceral injury is also supported by case reports of visceral injury after LDB-assisted CPR, including reported liver and splenic injury as well as liver laceration with hemoperitoneum [24,25]. However, such evidence should still be interpreted cautiously.

Considering evidence on clinical implications, recent survivor cohorts suggest that chest wall injuries are frequent and may be associated with clinically meaningful outcomes, underscoring the clinical relevance of systematic injury characterization rather than treating these findings as incidental [8,26]. The signal for liver lacerations in the LDB group also aligns with emerging attention to abdominal organ injury after cardiac arrest. Although less frequent than thoracic skeletal trauma, a recent systematic literature review highlights wide variability in reported rates of traumatic abdominal organ injury (predominantly liver and spleen) after cardiac arrest and emphasizes that even low-grade injuries can have management implications in the post-ROSC phase [27]. Against this background, identifying a device-associated difference would be relevant for risk awareness and post-resuscitation monitoring strategies, particularly in settings where early invasive procedures or anticoagulation may be considered. This work therefore adds evidence precisely at a point that is currently underrepresented: early CT-based injury assessment in a living post-ROSC cohort, with a direct comparison between different mechanical compression mechanisms.

The present study has several limitations that should be considered when interpreting the results. First, it has a retrospective single-center design and included a relatively small sample, which may have limited statistical power, particularly for rare complications. In addition, because multiple injury endpoints were analyzed without formal correction for multiple testing, type I error cannot be excluded. Second, the patient groups differed significantly in age. Advanced age is a recognized risk factor for a higher incidence of CPR-related injuries [28,29,30]. Therefore, the higher age in the LDB group may have contributed to the greater frequency of liver lacerations and displaced rib fractures observed in this cohort. Although both groups had a mean age above 55 years, residual confounding cannot be excluded. Third, additional unmeasured confounders may have influenced injury risk and injury detection, including CPR quality metrics, device positioning accuracy and device operator experience, transport conditions, and pharmacologic interventions. Retrospective documentation of resuscitation duration was incomplete in both groups, mainly owing to incomplete prehospital records. Given that all patients initially received manual CPR and were converted to mechanical compression only if sustained ROSC was not achieved, durations of <60 min or >120 min are unlikely. Accordingly, these considerations suggest that resuscitation duration was broadly comparable between groups. Furthermore, the exact prehospital decision process regarding device selection was not documented in a standardized manner. Device choice may therefore have been influenced by local EMS practice, device availability, or operator preference. Over the 6-year study period, temporal changes in local workflows and training may also have affected both device selection and injury patterns. Lastly, it needs to be acknowledged that early CT may theoretically underestimate some injuries because delayed complications, such as evolving pneumothorax or bleeding, may only become apparent later in the clinical course. In addition, the occurrence of injury-related complications and clinical significance of individual imaging findings were not systematically documented in this retrospective cohort and could therefore not be assessed reliably. Overall, these findings should therefore be interpreted as associative and hypothesis-generating rather than as proof of superiority of one device mechanism over another.

At the same time, the study also has several strengths. It was conducted independently of device manufacturers in a real-world CAC setting and directly compared two different mechanical compression mechanisms. In addition, injuries were assessed in a clinically relevant survivor cohort using a standardized early WBCT protocol, minimizing selection bias. All examinations were performed on the same CT scanner and independently reviewed by two blinded reviewers. The substantial inter-rater agreement further supports the internal consistency of the injury classification.

From a clinical perspective, the observed differences, particularly displaced rib fractures and liver lacerations, are relevant because they may affect post-resuscitation management. Chest wall injuries in survivors have been associated with clinically meaningful outcomes in contemporary cohorts, including respiratory complications and resource utilization [8,26,31]. Displaced rib fractures may theoretically complicate ventilation strategies and contribute to pulmonary morbidity, while abdominal organ injury, even if low-grade, can influence monitoring intensity and procedural decision-making, especially when antithrombotic therapy or invasive interventions are planned [27]. In practical terms, our findings support heightened awareness for abdominal injury and fracture displacement patterns when LDB devices are used. However, given the small cohort size and the observational, hypothesis-generating nature of our findings, device selection should continue to be based primarily on operational considerations and current guideline recommendations until larger studies confirm these results.

Future multicenter studies with prospective data collection, including device positioning parameters, CPR quality metrics, and clinically relevant outcome measures, would be valuable to clarify whether the observed device-associated injury signals persist after adjustment for confounding and to identify patient subgroups in whom one mechanism may be preferable.

## 5. Conclusions

In this CT-based cohort of resuscitated OHCA survivors, overall injury frequencies were broadly comparable between the two mechanical chest compression systems. However, the load-distributing band device was associated with a higher frequency of liver lacerations and displaced rib fractures than the piston-based device. These findings were derived from a retrospective single-center study with a limited sample size and should therefore be interpreted as associative and hypothesis-generating. Nevertheless, the present study adds clinically relevant evidence by comparing injury patterns between two different mechanical compression mechanisms in a living post-ROSC cohort assessed by early CT.

## Figures and Tables

**Table 1:** Characteristics of the Study Populations.

	Load-Distributing Band	Piston-Based	*p*-Value
Mean age (years)	62.81	55.41	0.03 ^1^
Male (*n*)/Female (*n*)	23/9	29/10	0.81 ^2^
Chest circumference (mm)	1115	1128	0.18 ^1^
Presternal adipose tissue (mm)	22	20	0.42 ^1^
CPR Duration ≥ 1 h (%) ^3,4^	68.42	63.33	0.71 ^2^

^1^ Calculated Using Mann–Whitney U-Test for continuous variables. ^2^ Calculated Using Chi-Square Test for categorical variables. ^3^ Relative proportion of patients who were resuscitated for over an hour. ^4^ Missing values for 13 patients in the load-distributing band group (40.61%) and 9 in the piston-based group (23.08%).

**Table 2:** Comparison of chest compression-related injuries.

	Load-Distributing Band (*n*)	Piston-Based (*n*)	χ ^1^	*p*-Value ^1^	Φ Phi ^2^
Active Bleeding	9 (28.1%)	8 (20.5%)	0.56	0.45	0.09
Liver laceration	5 (15.6%)	0 (0%)	6.55	0.01	0.30
Haemorrhagic pericardial effusion	5 (15.6%)	2 (5.1%)	2.18	0.14	0.17
Spleen laceration	0 (0%)	0 (0%)	-	-	-
Sternal fracture	25 (78.1%)	25 (64.1%)	1.66	0.20	0.15
Pneumothorax	13 (40.6%)	14 (35.9%)	0.17	0.68	0.05
Lung contusion	10 (31.3%)	8 (20.5%)	1.07	0.3	0.12
Mediastinal hematoma	15 (46.9%)	20 (51.3%)	0.14	0.71	0.04
Thoracic wall hematoma	9 (28.1%)	14 (35.9%)	0.48	0.49	0.08
Spine fracture	2 (6.3%)	2 (5.1%)	0.04	0.838	0.02

^1^ Chi-square test. ^2^ effect size (Phi): $\Phi =\sqrt{\frac{\chi^{2}}{N}}$ = w (0.1 ≤ w < 0.3 weak, 0.3 ≤ w < 0.5 moderate, w> 0.5 strong correlation).

**Table 3:** Comparison of chest compression-related rib fractures.

	Load-Distributing Band (*n*)	Piston-Based (*n*)	*p*-Value	MWU ^1^	Z ^2^	r ^3^
All rib fractures	300	339	0.43	556.5	0.78	0.09
Double rib fracture	39	39	0.33	551.0	0.97	0.11
Dislocated rib fracture	117	87	0.046	454.5	1.99	0.24
Non-dislocated rib fracture	85	115	0.75	597.0	0.32	0.03
Incomplete rib fracture	59	98	0.19	512.5	1.31	0.16

^1^ Mann–Whitney U-test. ^2^ Z-score. ^3^ correlation coefficient: $r=\left|\frac{Z}{\sqrt{n}}\right|$ (0.1 ≤ r < 0.3 weak, 0.3 ≤ r < 0.5 moderate, r > 0.5 strong correlation).

**Table 4:** Comparison of chest compression-related rib fractures on a patient level with corresponding risk ratios.

	Load-Distributing Band (*n* = 32)		Piston-Based (*n* = 39)		Risk Ratio	95%-CI	
	Patients (*n*)	Incidence ^1^ (%)	Patients (*n*)	Incidence ^1^ (%)		LL	UL
Rib fractures	30	93.8	34	87.2	1.07	0.93	1.25
Double rib fracture	14	43.8	13	33.3	1.31	0.73	2.37
Dislocated rib fracture	27	84.4	23	59	1.43	1.06	1.93
Non-dislocated rib fracture	27	84.4	30	76.9	1.1	0.87	1.38
Incomplete rib fracture	23	71.9	31	79.5	0.90	0.69	1.18

CI = Confidence Interval, LL = Lower Limit, UL = Upper limit. ^1^ Relative patient risk within the cohort.

## Data Availability

The data presented in this study are available on reasonable request from the corresponding author. The data are not publicly available due to privacy or ethical restrictions.

## Supplementary Materials

The following supporting information can be downloaded at: https://www.mdpi.com/article/10.3390/diagnostics16081179/s1, CT-Protocol.

## References

[1] Yan S., Gan Y., Jiang N., Wang R., Chen Y., Luo Z., Zong Q., Chen S., Lv C. (2020). The global survival rate among adult out-of-hospital cardiac arrest patients who received cardiopulmonary resuscitation: A systematic review and meta-analysis. Crit. Care.

[2] Gräsner J.T., Herlitz J., Tjelmeland I.B.M., Wnent J., Masterson S., Lilja G., Bein B., Böttiger B.W., Rosell-Ortiz F., Nolan J.P. (2021). European Resuscitation Council Guidelines 2021: Epidemiology of cardiac arrest in Europe. Resuscitation.

[3] MacDonald R.D. (2018). Articles That May Change Your Practice: Mechanical Chest Compressions. Air Med. J..

[4] Soar J., Böttiger B.W., Carli P., Jiménez F.C., Cimpoesu D., Cole G., Couper K., D’Arrigo S., Deakin C.D., Ek J.E. (2025). European Resuscitation Council Guidelines 2025 Adult Advanced Life Support. Resuscitation.

[5] Karatasakis A., Sarikaya B., Liu L., Gunn M.L., Kudenchuk P.J., Gatewood M.O., Maynard C., Sayre M.R., Counts C.R., Carlbom D.J. (2022). Prevalence and Patterns of Resuscitation-Associated Injury Detected by Head-to-Pelvis Computed Tomography After Successful Out-of-Hospital Cardiac Arrest Resuscitation. J. Am. Heart Assoc..

[6] Viniol S., Thomas R.P., König A.M., Betz S., Mahnken A.H. (2020). Early whole-body CT for treatment guidance in patients with return of spontaneous circulation after cardiac arrest. Emerg. Radiol..

[7] Tsuchida T., Kamiishi T., Usubuchi H., Semba A., Takahashi M., Mizugaki A., Hayamizu M., Hayakawa M., Wada T. (2024). Complication frequency of mechanical chest compression devices: A single-center, blinded study using retrospective data. Resusc. Plus.

[8] Prins J.T., Van Lieshout E.M., Van Wijck S.F.M., Scholte N.T., Den Uil C.A., Vermeulen J., Verhofstad M.H., Wijffels M.M. (2021). Chest wall injuries due to cardiopulmonary resuscitation and the effect on in-hospital outcomes in survivors of out-of-hospital cardiac arrest. J. Trauma. Acute Care Surg..

[9] Van Wijck S.F.M., Prins J.T.H., Verhofstad M.H.J., Wijffels M.M., Van Lieshout E.M. (2024). Rib fractures and other injuries after cardiopulmonary resuscitation for non-traumatic cardiac arrest: A systematic review and meta-analysis. Eur. J. Trauma. Emerg. Surg..

[10] Elm Evon Altman D.G., Egger M., Pocock S.J., Gøtzsche P.C., Vandenbroucke J.P. (2007). The Strengthening the Reporting of Observational Studies in Epidemiology (STROBE) Statement: Guidelines for Reporting Observational Studies. PLoS Med..

[11] Nolan J.P., Sandroni C., Böttiger B.W., Cariou A., Cronberg T., Friberg H., Genbrugge C., Haywood K., Lilja G., Moulaert V.R. (2021). European Resuscitation Council and European Society of Intensive Care Medicine Guidelines 2021: Post-resuscitation care. Resuscitation.

[12] Juskeviciute A., Resch M.A., Kumle B., Busch H.J., Janssens U., Michels G., Herda L.R., Faber M., Merz S., Reindl M. (2025). CT imaging in post-resuscitation care of non-traumatic resuscitation room patients in German hospitals. BMC Emerg. Med..

[13] Adel J., Akin M., Garcheva V., Vogel-Claussen J., Bauersachs J., Napp L.C., Schäfer A. (2022). Computed-Tomography as First-line Diagnostic Procedure in Patients With Out-of-Hospital Cardiac Arrest. Front. Cardiovasc. Med..

[14] Chien L.-C., Vakil M., Nguyen J., Chahine A., Archer-Arroyo K., Hanna T.N., Herr K.D. (2020). The American Association for the Surgery of Trauma Organ Injury Scale 2018 update for computed tomography-based grading of renal trauma: A primer for the emergency radiologist. Emerg. Radiol..

[15] Landis J.R., Koch G.G. (1977). The Measurement of Observer Agreement for Categorical Data. Biometrics.

[16] Kim H., Yoon S.Y., Han J., Seok J., Kang W.S. (2025). Non-Completely Displaced Traumatic Rib Fractures: Potentially Less Crucial for Pulmonary Adverse Outcomes, Regardless of Classification. Medicina.

[17] Preda T., Nafi M., Villa M., Cassina T. (2023). Traumatic injuries after manual and automatic mechanical compression during cardiopulmonary resuscitation, a retrospective cohort study. Resusc. Plus.

[18] Eichhorn S., Mendoza Garcia A., Polski M., Spindler J., Stroh A., Heller M., Lange R., Krane M. (2017). Corpuls cpr resuscitation device generates superior emulated flows and pressures than LUCAS II in a mechanical thorax model. Australas. Phys. Eng. Sci. Med..

[19] Kim H.T., Kim J.G., Jang Y.S., Kang G.H., Kim W., Choi H.Y., Jun G.S. (2019). Comparison of in-hospital use of mechanical chest compression devices for out-of-hospital cardiac arrest patients. Medicine.

[20] Wik L., Olsen J.-A., Persse D., Sterz F., Lozano M., Brouwer M.A., Westfall M., Souders C.M., Malzer R., van Grunsven P.M. (2014). Manual vs. integrated automatic load-distributing band CPR with equal survival after out of hospital cardiac arrest. The randomized CIRC trial. Resuscitation.

[21] Perkins G.D., Lall R., Quinn T., Deakin C.D., Cooke M.W., Horton J., Lamb S.E., Slowther A.M., Woollard M., Carson A. (2015). Mechanical versus manual chest compression for out-of-hospital cardiac arrest (PARAMEDIC): A pragmatic, cluster randomised controlled trial. Lancet.

[22] Viniol S., Thomas R.P., Gombert S., König A.M., Betz S., Mahnken A.H. (2020). Comparison of different resuscitation methods with regard to injury patterns in cardiac arrest survivors based on computer tomography. Eur. J. Radiol..

[23] Koster R.W., Beenen L.F., van der Boom E.B., Spijkerboer A.M., Tepaske R., van der Wal A.C., Beesems S.G., Tijssen J.G. (2017). Safety of mechanical chest compression devices AutoPulse and LUCAS in cardiac arrest: A randomized clinical trial for non-inferiority. Eur. Heart J..

[24] Wind J., Bekkers S.C., van Hooren L.J., van Heurn L.W. (2009). Extensive injury after use of a mechanical cardiopulmonary resuscitation device. Am. J. Emerg. Med..

[25] Cho M.-J. (2024). Liver laceration as a post-cardiopulmonary resuscitation complication in a person with breast implants: A case report. J. Trauma Inj..

[26] Nemlaghi-Zdiri M., Lacoste-Palasset T., Dahmani S., Lefevre T., Haudebourg L., Voicu S., M’rad A., Lehalleur A.P., Sutterlin L., Ekherian J.M. (2025). Impact of chest wall injuries after cardiopulmonary resuscitation in non-traumatic and successfully resuscitated cardiac arrest: Insights from the DISCAR study. Resuscitation.

[27] Hoftun Farbu B., Hagemo J., Rehn M. (2025). Abdominal organ injury in cardiac arrest: Systematic literature review. PLoS ONE.

[28] Setälä P., Hellevuo H., Huhtala H., Kämäräinen A., Tirkkonen J., Hoppu S. (2018). Risk factors for cardiopulmonary resuscitation-related injuries sustained during out-of-hospital cardiac arrests. Acta Anaesthesiol. Scand..

[29] Takayama W., Koguchi H., Endo A., Otomo Y. (2018). The Association between Cardiopulmonary Resuscitation in Out-of-Hospital Settings and Chest Injuries: A Retrospective Observational Study. Prehospital Disaster Med..

[30] Moriguchi S., Hamanaka K., Nakamura M., Takaso M., Baba M., Hitosugi M. (2021). Aging is only significant factor causing CPR-induced injuries and serious injuries. Leg. Med..

[31] Al Nouh M., Caragounis E.-C., Rossi Norrlund R., Fagevik Olsén M. (2024). Favourable outcome in survivors of CPR-related chest wall injuries. Injury.

