# Supplementary material for: Injury Patterns in Resuscitated Non-Traumatic Cardiac Arrest Patients—A Comparative CT Analysis Between Automated Chest Compression Devices
Source: Diagnostics (Basel). 2026 Apr 16;16(8):1179. doi: 10.3390/diagnostics16081179 (PMC13115485; doi:10.3390/diagnostics16081179)
Supplement: Supplementary file 1 [file diagnostics-16-01179-s001.zip › diagnostics-4203528-supplementary.pdf]

CT-Protocol:

All included patients were examined within 6 hours after admission using a 64-slice detector row CT scanner (Definition AS®, Siemens Healthineers, Forchheim, Germany) using a standardized CT protocol. The protocol included a non-enhanced cranial CT, a CT angiography from the head to the groin and 30 seconds thereafter a venous phase CT of the abdomen. Every patient received 110 ml intravenous contrast agent (Ultravist 370®, Bayer, Leverkusen, Germany) followed by saline chaser bolus of 30 ml with a flow rate of 4 ml/s. The assessment of resuscitation-related injuries was conducted using a 2 mm axial reconstruction (B30F kernel, W/L: 150/700 HF), a 3 mm axial reconstruction (B70F Kernel, W/L: -500/1600 HF), a 3 mm axial reconstruction (B30F Kernel, W/L 50/320 HF), a 3 mm sagittal reconstruction (B70F Kernel, W/L: 450/1500 HF) and 3 mm coronal reconstruction (B31F Kernel, W/L: 50/320 HF).
